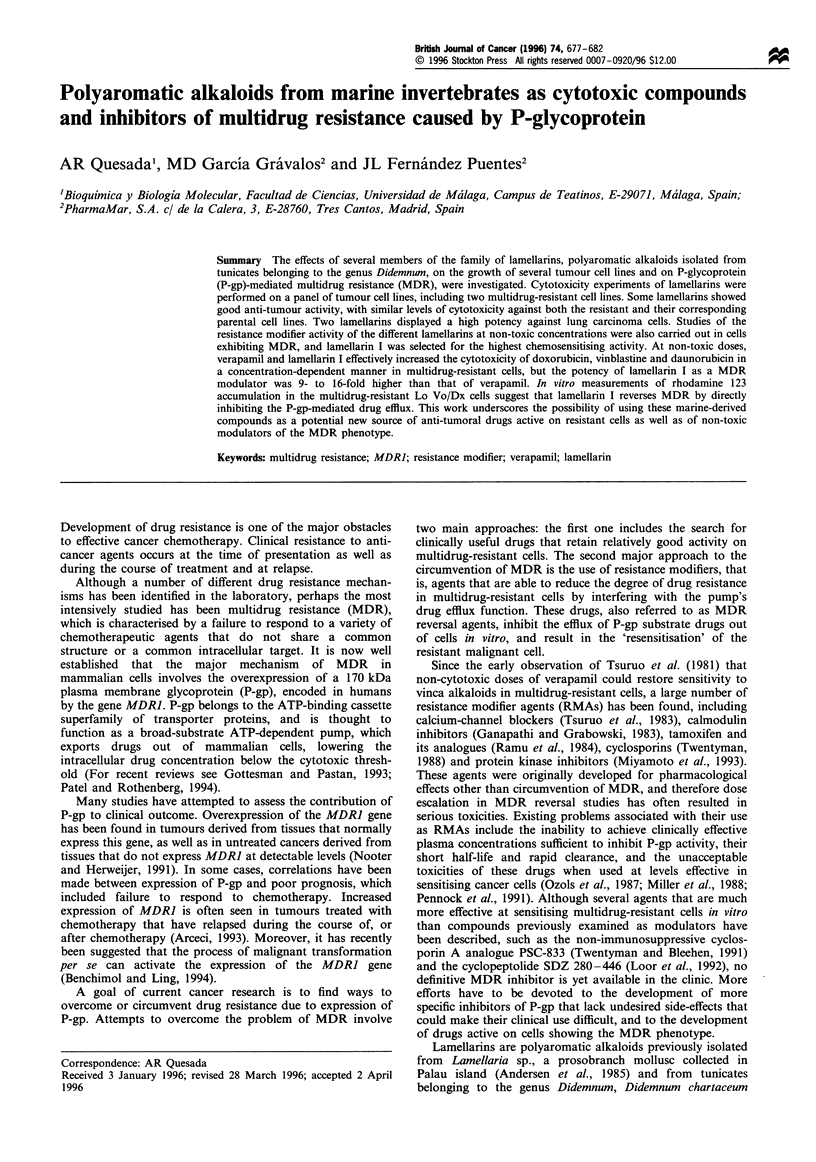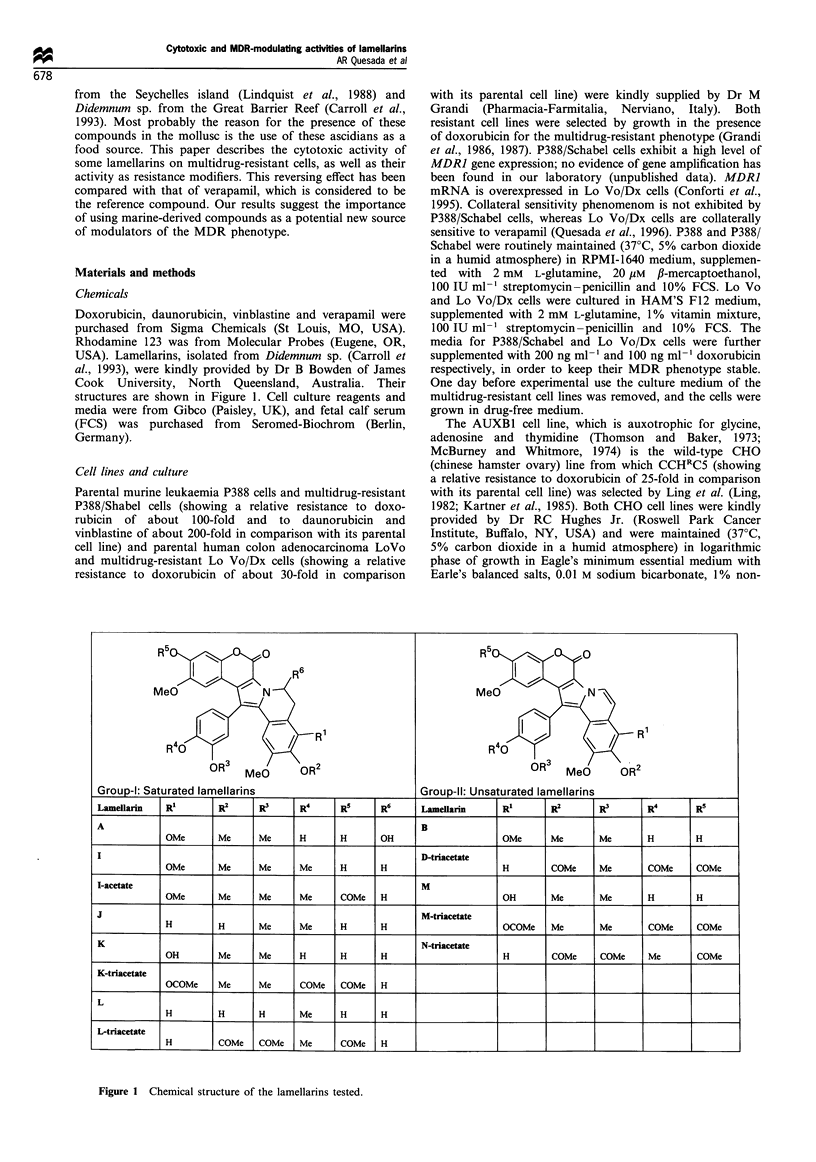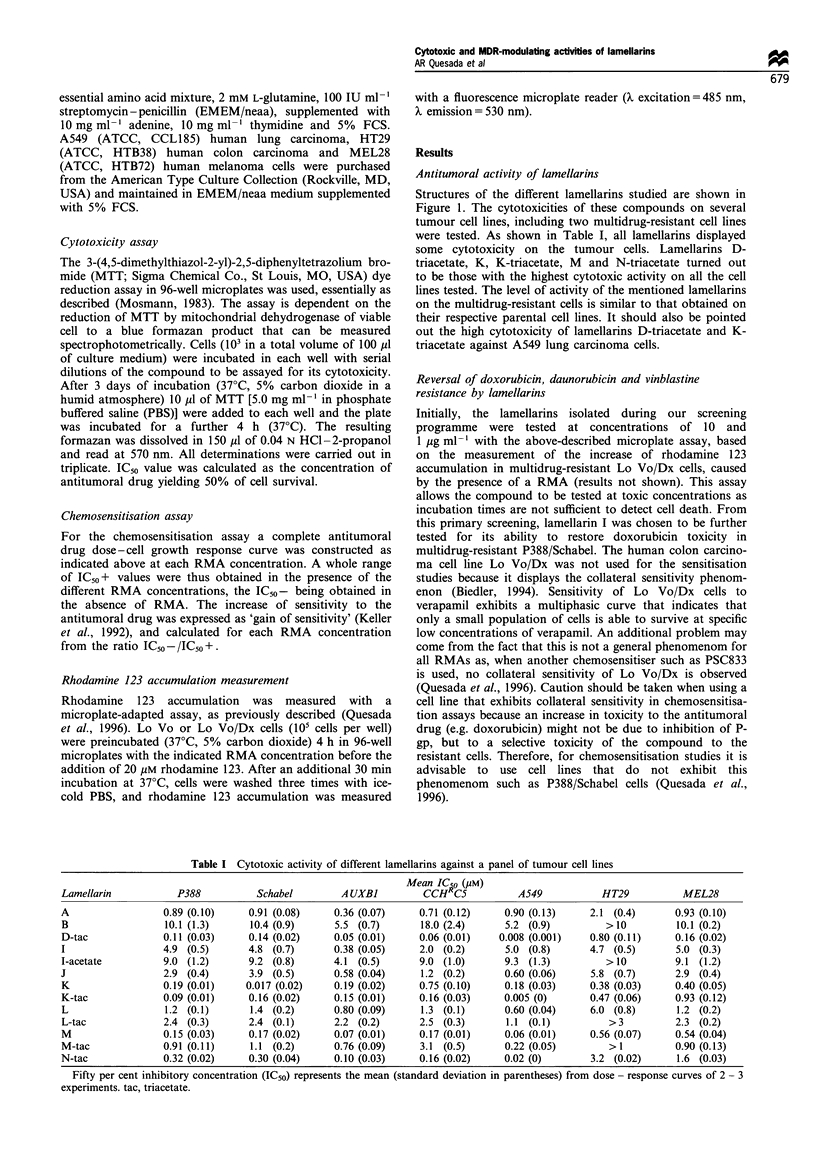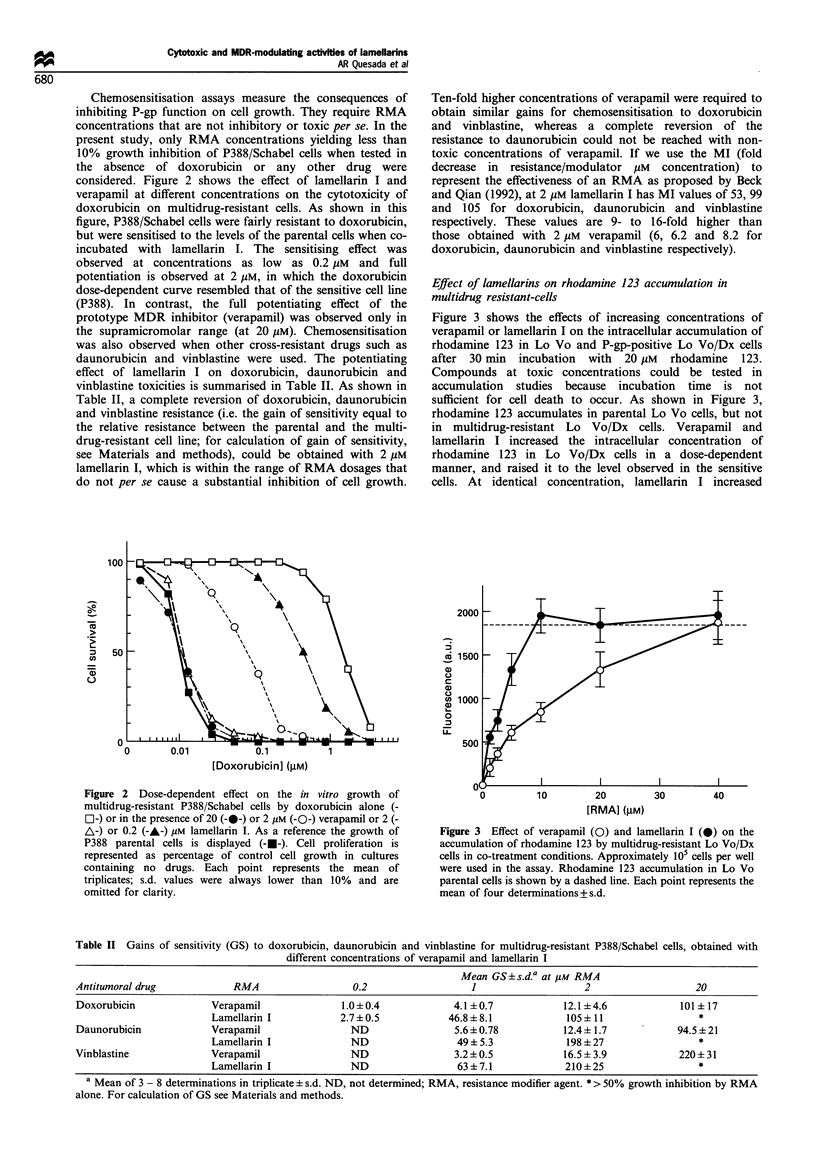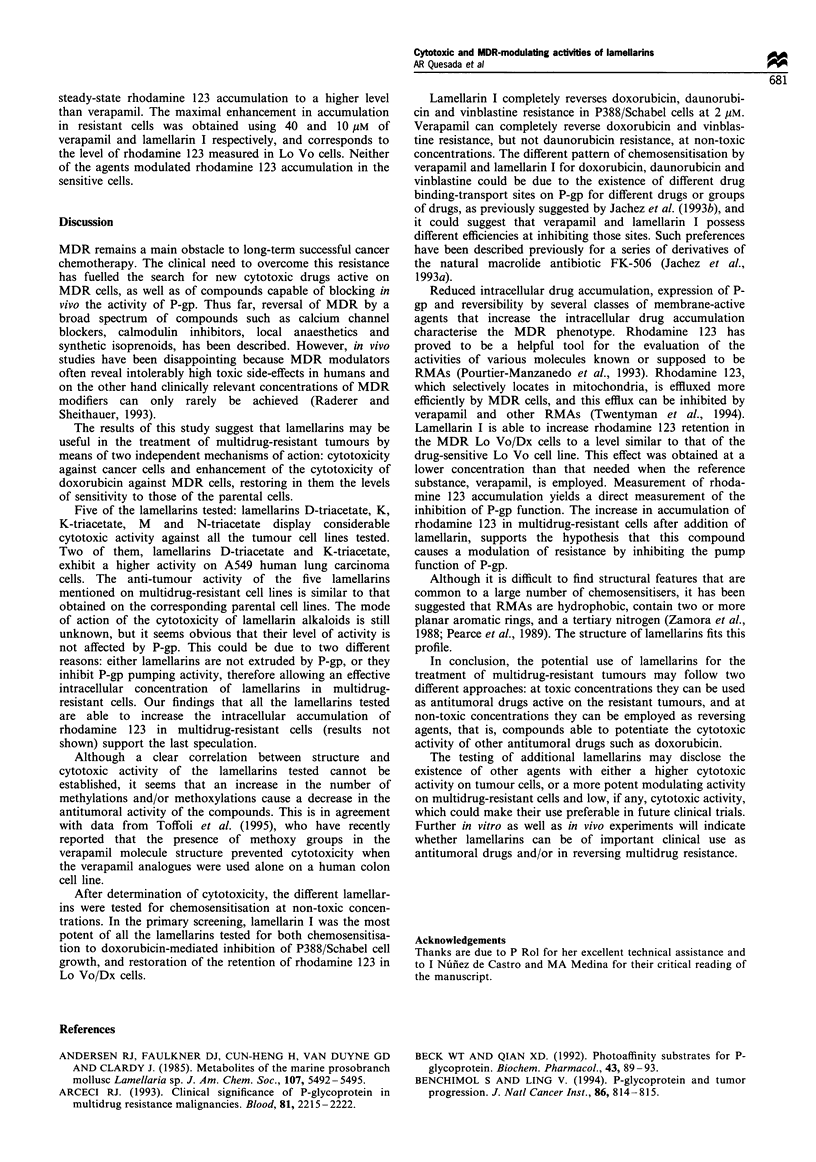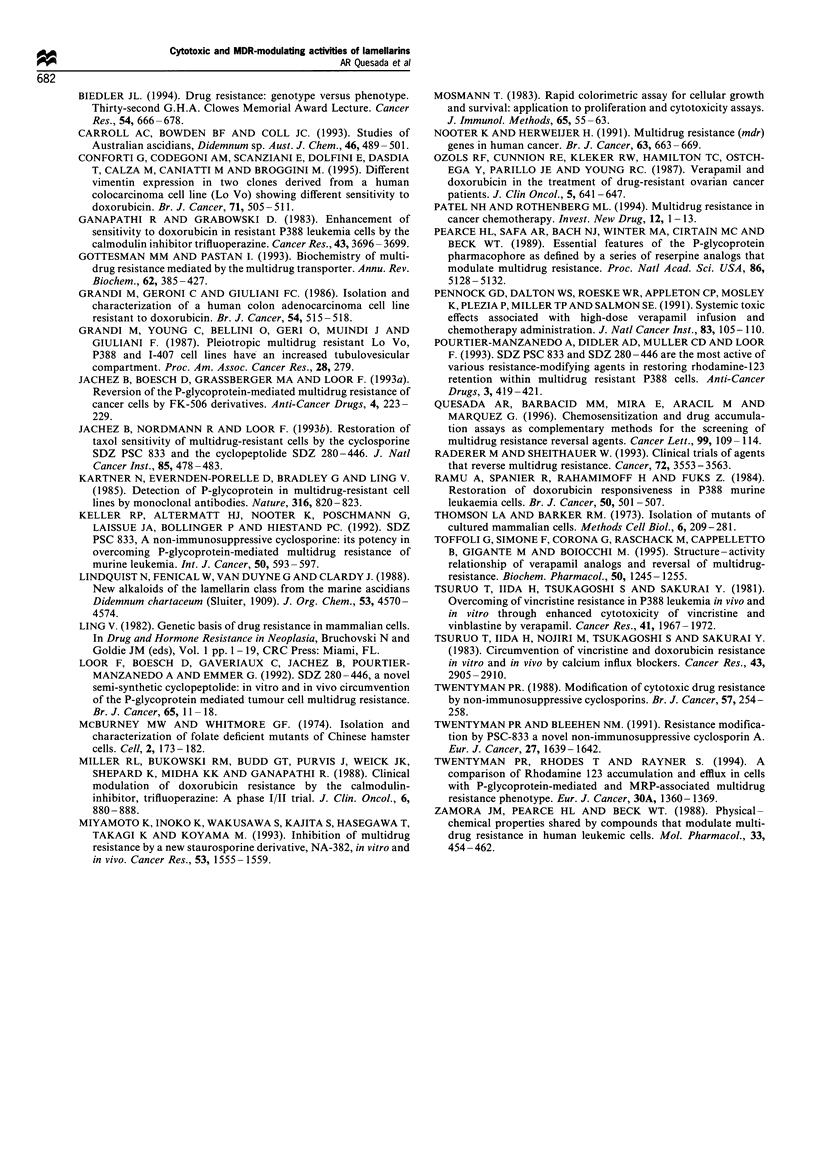# Polyaromatic alkaloids from marine invertebrates as cytotoxic compounds and inhibitors of multidrug resistance caused by P-glycoprotein.

**DOI:** 10.1038/bjc.1996.421

**Published:** 1996-09

**Authors:** A. R. Quesada, M. D. García Grávalos, J. L. Fernández Puentes

**Affiliations:** Facultad de Ciencias, Universidad de Málaga, Spain.

## Abstract

The effects of several members of the family of lamellarins, polyaromatic alkaloids isolated from tunicates belonging to the genus Didemnum, on the growth of several tumour cell lines and on P-glycoprotein (P-gp)-mediated multidrug resistance (MDR), were investigated. Cytotoxicity experiments of lamellarins were performed on a panel of tumour cell lines, including two multidrug-resistant cell lines. Some lamellarins showed good anti-tumour activity, with similar levels of cytotoxicity against both the resistant and their corresponding parental cell lines. Two lamellarins displayed a high potency against lung carcinoma cells. Studies of the resistance modifier activity of the different lamellarins at non-toxic concentrations were also carried out in cells exhibiting MDR, and lamellarin I was selected for the highest chemosensitising activity. At non-toxic doses, verapamil and lamellarin I effectively increased the cytotoxicity of doxorubicin, vinblastine and daunorubicin in a concentration-dependent manner in multidrug-resistant cells, but the potency of lamellarin I as a MDR modulator was 9- to 16-fold higher than that of verapamil. In vitro measurements of rhodamine 123 accumulation in the multidrug-resistant Lo Vo/Dx cells suggest that lamellarin I reverses MDR by directly inhibiting the P-gp-mediated drug efflux. This work underscores the possibility of using these marine-derived compounds as a potential new source of anti-tumoral drugs active on resistant cells as well as of non-toxic modulators of the MDR phenotype.


British Journal of Cancer (1996) 74, 677-682
© 1996 Stockton Press All rights reserved 0007-0920/96 $12.00

### Polyaromatic alkaloids from marine invertebrates as cytotoxic compounds and inhibitors of multidrug resistance caused by P-glycoprotein

AR Quesada[1], MD García Grávalos[2] and JL Fernández Puentes[2]

[1]*Bioquímica y Biología Molecular, Facultad de Ciencias, Universidad de Málaga, Campus de Teatinos, E-29071, Málaga, Spain;* [2]*PharmaMar, S.A. c/ de la Calera, 3, E-28760, Tres Cantos, Madrid, Spain*

**Summary** The effects of several members of the family of lamellarins, polyaromatic alkaloids isolated from tunicates belonging to the genus *Didemnum*, on the growth of several tumour cell lines and on P-glycoprotein (P-gp)-mediated multidrug resistance (MDR), were investigated. Cytotoxicity experiments of lamellarins were performed on a panel of tumour cell lines, including two multidrug-resistant cell lines. Some lamellarins showed good anti-tumour activity, with similar levels of cytotoxicity against both the resistant and their corresponding parental cell lines. Two lamellarins displayed a high potency against lung carcinoma cells. Studies of the resistance modifier activity of the different lamellarins at non-toxic concentrations were also carried out in cells exhibiting MDR, and lamellarin I was selected for the highest chemosensitising activity. At non-toxic doses, verapamil and lamellarin I effectively increased the cytotoxicity of doxorubicin, vinblastine and daunorubicin in a concentration-dependent manner in multidrug-resistant cells, but the potency of lamellarin I as a MDR modulator was 9- to 16-fold higher than that of verapamil. *In vitro* measurements of rhodamine 123 accumulation in the multidrug-resistant Lo Vo/Dx cells suggest that lamellarin I reverses MDR by directly inhibiting the P-gp-mediated drug efflux. This work underscores the possibility of using these marine-derived compounds as a potential new source of anti-tumoral drugs active on resistant cells as well as of non-toxic modulators of the MDR phenotype.

**Keywords:** multidrug resistance; *MDR1*; resistance modifier; verapamil; lamellarin

Development of drug resistance is one of the major obstacles to effective cancer chemotherapy. Clinical resistance to anti-cancer agents occurs at the time of presentation as well as during the course of treatment and at relapse.

Although a number of different drug resistance mechanisms has been identified in the laboratory, perhaps the most intensively studied has been multidrug resistance (MDR), which is characterised by a failure to respond to a variety of chemotherapeutic agents that do not share a common structure or a common intracellular target. It is now well established that the major mechanism of MDR in mammalian cells involves the overexpression of a 170 kDa plasma membrane glycoprotein (P-gp), encoded in humans by the gene *MDR1*. P-gp belongs to the ATP-binding cassette superfamily of transporter proteins, and is thought to function as a broad-substrate ATP-dependent pump, which exports drugs out of mammalian cells, lowering the intracellular drug concentration below the cytotoxic threshold (For recent reviews see Gottesman and Pastan, 1993; Patel and Rothenberg, 1994).

Many studies have attempted to assess the contribution of P-gp to clinical outcome. Overexpression of the *MDR1* gene has been found in tumours derived from tissues that normally express this gene, as well as in untreated cancers derived from tissues that do not express *MDR1* at detectable levels (Nooter and Herweijer, 1991). In some cases, correlations have been made between expression of P-gp and poor prognosis, which included failure to respond to chemotherapy. Increased expression of *MDR1* is often seen in tumours treated with chemotherapy that have relapsed during the course of, or after chemotherapy (Arceci, 1993). Moreover, it has recently been suggested that the process of malignant transformation *per se* can activate the expression of the *MDR1* gene (Benchimol and Ling, 1994).

A goal of current cancer research is to find ways to overcome or circumvent drug resistance due to expression of P-gp. Attempts to overcome the problem of MDR involve two main approaches: the first one includes the search for clinically useful drugs that retain relatively good activity on multidrug-resistant cells. The second major approach to the circumvention of MDR is the use of resistance modifiers, that is, agents that are able to reduce the degree of drug resistance in multidrug-resistant cells by interfering with the pump's drug efflux function. These drugs, also referred to as MDR reversal agents, inhibit the efflux of P-gp substrate drugs out of cells *in vitro*, and result in the 'resensitisation' of the resistant malignant cell.

Since the early observation of Tsuruo *et al.* (1981) that non-cytotoxic doses of verapamil could restore sensitivity to vinca alkaloids in multidrug-resistant cells, a large number of resistance modifier agents (RMAs) has been found, including calcium-channel blockers (Tsuruo *et al.*, 1983), calmodulin inhibitors (Ganapathi and Grabowski, 1983), tamoxifen and its analogues (Ramu *et al.*, 1984), cyclosporins (Twentyman, 1988) and protein kinase inhibitors (Miyamoto *et al.*, 1993). These agents were originally developed for pharmacological effects other than circumvention of MDR, and therefore dose escalation in MDR reversal studies has often resulted in serious toxicities. Existing problems associated with their use as RMAs include the inability to achieve clinically effective plasma concentrations sufficient to inhibit P-gp activity, their short half-life and rapid clearance, and the unacceptable toxicities of these drugs when used at levels effective in sensitising cancer cells (Ozols *et al.*, 1987; Miller *et al.*, 1988; Pennock *et al.*, 1991). Although several agents that are much more effective at sensitising multidrug-resistant cells *in vitro* than compounds previously examined as modulators have been described, such as the non-immunosuppressive cyclosporin A analogue PSC-833 (Twentyman and Bleehen, 1991) and the cyclopeptolide SDZ 280-446 (Loor *et al.*, 1992), no definitive MDR inhibitor is yet available in the clinic. More efforts have to be devoted to the development of more specific inhibitors of P-gp that lack undesired side-effects that could make their clinical use difficult, and to the development of drugs active on cells showing the MDR phenotype.

Lamellarins are polyaromatic alkaloids previously isolated from *Lamellaria* sp., a prosobranch mollusc collected in Palau island (Andersen *et al.*, 1985) and from tunicates belonging to the genus *Didemnum*, *Didemnum chartaceum*

Correspondence: AR Quesada
Received 3 January 1996; revised 28 March 1996; accepted 2 April 1996

from the Seychelles island (Lindquist *et al.*, 1988) and *Didemnum* sp. from the Great Barrier Reef (Carroll *et al.*, 1993). Most probably the reason for the presence of these compounds in the mollusc is the use of these ascidians as a food source. This paper describes the cytotoxic activity of some lamellarins on multidrug-resistant cells, as well as their activity as resistance modifiers. This reversing effect has been compared with that of verapamil, which is considered to be the reference compound. Our results suggest the importance of using marine-derived compounds as a potential new source of modulators of the MDR phenotype.

### Materials and methods

### *Chemicals*

Doxorubicin, daunorubicin, vinblastine and verapamil were purchased from Sigma Chemicals (St Louis, MO, USA). Rhodamine 123 was from Molecular Probes (Eugene, OR, USA). Lamellarins, isolated from *Didemnum* sp. (Carroll *et al.*, 1993), were kindly provided by Dr B Bowden of James Cook University, North Queensland, Australia. Their structures are shown in Figure 1. Cell culture reagents and media were from Gibco (Paisley, UK), and fetal calf serum (FCS) was purchased from Seromed-Biochrom (Berlin, Germany).

### *Cell lines and culture*

Parental murine leukaemia P388 cells and multidrug-resistant P388/Shabel cells (showing a relative resistance to doxorubicin of about 100-fold and to daunorubicin and vinblastine of about 200-fold in comparison with its parental cell line) and parental human colon adenocarcinoma LoVo and multidrug-resistant Lo Vo/Dx cells (showing a relative resistance to doxorubicin of about 30-fold in comparison with its parental cell line) were kindly supplied by Dr M Grandi (Pharmacia-Farmitalia, Nerviano, Italy). Both resistant cell lines were selected by growth in the presence of doxorubicin for the multidrug-resistant phenotype (Grandi *et al.*, 1986, 1987). P388/Schabel cells exhibit a high level of *MDR1* gene expression; no evidence of gene amplification has been found in our laboratory (unpublished data). *MDR1* mRNA is overexpressed in Lo Vo/Dx cells (Conforti *et al.*, 1995). Collateral sensitivity phenomenom is not exhibited by P388/Schabel cells, whereas Lo Vo/Dx cells are collaterally sensitive to verapamil (Quesada *et al.*, 1996). P388 and P388/Schabel were routinely maintained (37°C, 5% carbon dioxide in a humid atmosphere) in RPMI-1640 medium, supplemented with 2 mM L-glutamine, 20 $\mu$M $\beta$-mercaptoethanol, 100 IU $ml^{-1}$ streptomycin–penicillin and 10% FCS. Lo Vo and Lo Vo/Dx cells were cultured in HAM'S F12 medium, supplemented with 2 mM L-glutamine, 1% vitamin mixture, 100 IU $ml^{-1}$ streptomycin–penicillin and 10% FCS. The media for P388/Schabel and Lo Vo/Dx cells were further supplemented with 200 ng $ml^{-1}$ and 100 ng $ml^{-1}$ doxorubicin respectively, in order to keep their MDR phenotype stable. One day before experimental use the culture medium of the multidrug-resistant cell lines was removed, and the cells were grown in drug-free medium.

The AUXB1 cell line, which is auxotrophic for glycine, adenosine and thymidine (Thomson and Baker, 1973; McBurney and Whitmore, 1974) is the wild-type CHO (chinese hamster ovary) line from which CCH$^R$C5 (showing a relative resistance to doxorubicin of 25-fold in comparison with its parental cell line) was selected by Ling *et al.* (Ling, 1982; Kartner *et al.*, 1985). Both CHO cell lines were kindly provided by Dr RC Hughes Jr. (Roswell Park Cancer Institute, Buffalo, NY, USA) and were maintained (37°C, 5% carbon dioxide in a humid atmosphere) in logarithmic phase of growth in Eagle's minimum essential medium with Earle's balanced salts, 0.01 M sodium bicarbonate, 1% non-

Group-I: Saturated lamellarins

| Lamellarin | R1 | R2 | R3 | R4 | R5 | R6 |
|---|---|---|---|---|---|---|
| A | OMe | Me | Me | H | H | OH |
| I | OMe | Me | Me | Me | H | H |
| I-acetate | OMe | Me | Me | Me | COMe | H |
| J | H | H | Me | Me | H | H |
| K | OH | Me | Me | H | H | H |
| K-triacetate | OCOMe | Me | Me | COMe | COMe | H |
| L | H | H | H | Me | H | H |
| L-triacetate | H | COMe | COMe | Me | COMe | H |

Group-II: Unsaturated lamellarins

| Lamellarin | R1 | R2 | R3 | R4 | R5 |
|---|---|---|---|---|---|
| B | OMe | Me | Me | H | H |
| D-triacetate | H | COMe | Me | COMe | COMe |
| M | OH | Me | Me | H | H |
| M-triacetate | OCOMe | Me | Me | COMe | COMe |
| N-triacetate | H | COMe | COMe | Me | COMe |

**Figure 1** Chemical structure of the lamellarins tested.

essential amino acid mixture, 2 mM L-glutamine, 100 IU $ml^{-1}$ streptomycin-penicillin (EMEM/neaa), supplemented with 10 mg $ml^{-1}$ adenine, 10 mg $ml^{-1}$ thymidine and 5% FCS. A549 (ATCC, CCL185) human lung carcinoma, HT29 (ATCC, HTB38) human colon carcinoma and MEL28 (ATCC, HTB72) human melanoma cells were purchased from the American Type Culture Collection (Rockville, MD, USA) and maintained in EMEM/neaa medium supplemented with 5% FCS.

### *Cytotoxicity assay*

The 3-(4,5-dimethylthiazol-2-yl)-2,5-diphenyltetrazolium bromide (MTT; Sigma Chemical Co., St Louis, MO, USA) dye reduction assay in 96-well microplates was used, essentially as described (Mosmann, 1983). The assay is dependent on the reduction of MTT by mitochondrial dehydrogenase of viable cell to a blue formazan product that can be measured spectrophotometrically. Cells ($10^3$ in a total volume of 100 $\mu l$ of culture medium) were incubated in each well with serial dilutions of the compound to be assayed for its cytotoxicity. After 3 days of incubation (37°C, 5% carbon dioxide in a humid atmosphere) 10 $\mu l$ of MTT [5.0 mg $ml^{-1}$ in phosphate buffered saline (PBS)] were added to each well and the plate was incubated for a further 4 h (37°C). The resulting formazan was dissolved in 150 $\mu l$ of 0.04 N HCl-2-propanol and read at 570 nm. All determinations were carried out in triplicate. $IC_{50}$ value was calculated as the concentration of antitumoral drug yielding 50% of cell survival.

### *Chemosensitisation assay*

For the chemosensitisation assay a complete antitumoral drug dose-cell growth response curve was constructed as indicated above at each RMA concentration. A whole range of $IC_{50}+$ values were thus obtained in the presence of the different RMA concentrations, the $IC_{50}-$ being obtained in the absence of RMA. The increase of sensitivity to the antitumoral drug was expressed as 'gain of sensitivity' (Keller *et al.*, 1992), and calculated for each RMA concentration from the ratio $IC_{50}-/IC_{50}+$.

### *Rhodamine 123 accumulation measurement*

Rhodamine 123 accumulation was measured with a microplate-adapted assay, as previously described (Quesada *et al.*, 1996). Lo Vo or Lo Vo/Dx cells ($10^5$ cells per well) were preincubated (37°C, 5% carbon dioxide) 4 h in 96-well microplates with the indicated RMA concentration before the addition of 20 $\mu$M rhodamine 123. After an additional 30 min incubation at 37°C, cells were washed three times with ice-cold PBS, and rhodamine 123 accumulation was measured with a fluorescence microplate reader ($\lambda$ excitation = 485 nm, $\lambda$ emission = 530 nm).

### Results

### *Antitumoral activity of lamellarins*

Structures of the different lamellarins studied are shown in Figure 1. The cytotoxicities of these compounds on several tumour cell lines, including two multidrug-resistant cell lines were tested. As shown in Table I, all lamellarins displayed some cytotoxicity on the tumour cells. Lamellarins D-triacetate, K, K-triacetate, M and N-triacetate turned out to be those with the highest cytotoxic activity on all the cell lines tested. The level of activity of the mentioned lamellarins on the multidrug-resistant cells is similar to that obtained on their respective parental cell lines. It should also be pointed out the high cytotoxicity of lamellarins D-triacetate and K-triacetate against A549 lung carcinoma cells.

### *Reversal of doxorubicin, daunorubicin and vinblastine resistance by lamellarins*

Initially, the lamellarins isolated during our screening programme were tested at concentrations of 10 and 1 $\mu g$ $ml^{-1}$ with the above-described microplate assay, based on the measurement of the increase of rhodamine 123 accumulation in multidrug-resistant Lo Vo/Dx cells, caused by the presence of a RMA (results not shown). This assay allows the compound to be tested at toxic concentrations as incubation times are not sufficient to detect cell death. From this primary screening, lamellarin I was chosen to be further tested for its ability to restore doxorubicin toxicity in multidrug-resistant P388/Schabel. The human colon carcinoma cell line Lo Vo/Dx was not used for the sensitisation studies because it displays the collateral sensitivity phenomenon (Biedler, 1994). Sensitivity of Lo Vo/Dx cells to verapamil exhibits a multiphasic curve that indicates that only a small population of cells is able to survive at specific low concentrations of verapamil. An additional problem may come from the fact that this is not a general phenomenom for all RMAs as, when another chemosensitiser such as PSC833 is used, no collateral sensitivity of Lo Vo/Dx is observed (Quesada *et al.*, 1996). Caution should be taken when using a cell line that exhibits collateral sensitivity in chemosensitisation assays because an increase in toxicity to the antitumoral drug (e.g. doxorubicin) might not be due to inhibition of P-gp, but to a selective toxicity of the compound to the resistant cells. Therefore, for chemosensitisation studies it is advisable to use cell lines that do not exhibit this phenomenom such as P388/Schabel cells (Quesada *et al.*, 1996).

**Table I** Cytotoxic activity of different lamellarins against a panel of tumour cell lines

| | Mean $IC_{50}$ ($\mu$M) | | | | | | |
|---|---|---|---|---|---|---|---|
| *Lamellarin* | *P388* | *Schabel* | *AUXB1* | *$CCH^RC5$* | *A549* | *HT29* | *MEL28* |
| A | 0.89 (0.10) | 0.91 (0.08) | 0.36 (0.07) | 0.71 (0.12) | 0.90 (0.13) | 2.1 (0.4) | 0.93 (0.10) |
| B | 10.1 (1.3) | 10.4 (0.9) | 5.5 (0.7) | 18.0 (2.4) | 5.2 (0.9) | >10 | 10.1 (0.2) |
| D-tac | 0.11 (0.03) | 0.14 (0.02) | 0.05 (0.01) | 0.06 (0.01) | 0.008 (0.001) | 0.80 (0.11) | 0.16 (0.02) |
| I | 4.9 (0.5) | 4.8 (0.7) | 0.38 (0.05) | 2.0 (0.2) | 5.0 (0.8) | 4.7 (0.5) | 5.0 (0.3) |
| I-acetate | 9.0 (1.2) | 9.2 (0.8) | 4.1 (0.5) | 9.0 (1.0) | 9.3 (1.3) | >10 | 9.1 (1.2) |
| J | 2.9 (0.4) | 3.9 (0.5) | 0.58 (0.04) | 1.2 (0.2) | 0.60 (0.06) | 5.8 (0.7) | 2.9 (0.4) |
| K | 0.19 (0.01) | 0.017 (0.02) | 0.19 (0.02) | 0.75 (0.10) | 0.18 (0.03) | 0.38 (0.03) | 0.40 (0.05) |
| K-tac | 0.09 (0.01) | 0.16 (0.02) | 0.15 (0.01) | 0.16 (0.03) | 0.005 (0) | 0.47 (0.06) | 0.93 (0.12) |
| L | 1.2 (0.1) | 1.4 (0.2) | 0.80 (0.09) | 1.3 (0.1) | 0.60 (0.04) | 6.0 (0.8) | 1.2 (0.2) |
| L-tac | 2.4 (0.3) | 2.4 (0.1) | 2.2 (0.2) | 2.5 (0.3) | 1.1 (0.1) | >3 | 2.3 (0.2) |
| M | 0.15 (0.03) | 0.17 (0.02) | 0.07 (0.01) | 0.17 (0.01) | 0.06 (0.01) | 0.56 (0.07) | 0.54 (0.04) |
| M-tac | 0.91 (0.11) | 1.1 (0.2) | 0.76 (0.09) | 3.1 (0.5) | 0.22 (0.05) | >1 | 0.90 (0.13) |
| N-tac | 0.32 (0.02) | 0.30 (0.04) | 0.10 (0.03) | 0.16 (0.02) | 0.02 (0) | 3.2 (0.02) | 1.6 (0.03) |

Fifty per cent inhibitory concentration ($IC_{50}$) represents the mean (standard deviation in parentheses) from dose – response curves of 2 – 3 experiments. tac, triacetate.

Chemosensitisation assays measure the consequences of inhibiting P-gp function on cell growth. They require RMA concentrations that are not inhibitory or toxic *per se*. In the present study, only RMA concentrations yielding less than 10% growth inhibition of P388/Schabel cells when tested in the absence of doxorubicin or any other drug were considered. Figure 2 shows the effect of lamellarin I and verapamil at different concentrations on the cytotoxicity of doxorubicin on multidrug-resistant cells. As shown in this figure, P388/Schabel cells were fairly resistant to doxorubicin, but were sensitised to the levels of the parental cells when co-incubated with lamellarin I. The sensitising effect was observed at concentrations as low as 0.2 $\mu$M and full potentiation is observed at 2 $\mu$M, in which the doxorubicin dose-dependent curve resembled that of the sensitive cell line (P388). In contrast, the full potentiating effect of the prototype MDR inhibitor (verapamil) was observed only in the supramicromolar range (at 20 $\mu$M). Chemosensitisation was also observed when other cross-resistant drugs such as daunorubicin and vinblastine were used. The potentiating effect of lamellarin I on doxorubicin, daunorubicin and vinblastine toxicities is summarised in Table II. As shown in Table II, a complete reversion of doxorubicin, daunorubicin and vinblastine resistance (i.e. the gain of sensitivity equal to the relative resistance between the parental and the multidrug-resistant cell line; for calculation of gain of sensitivity, see Materials and methods), could be obtained with 2 $\mu$M lamellarin I, which is within the range of RMA dosages that do not *per se* cause a substantial inhibition of cell growth. Ten-fold higher concentrations of verapamil were required to obtain similar gains for chemosensitisation to doxorubicin and vinblastine, whereas a complete reversion of the resistance to daunorubicin could not be reached with non-toxic concentrations of verapamil. If we use the MI (fold decrease in resistance/modulator $\mu$M concentration) to represent the effectiveness of an RMA as proposed by Beck and Qian (1992), at 2 $\mu$M lamellarin I has MI values of 53, 99 and 105 for doxorubicin, daunorubicin and vinblastine respectively. These values are 9- to 16-fold higher than those obtained with 2 $\mu$M verapamil (6, 6.2 and 8.2 for doxorubicin, daunorubicin and vinblastine respectively).

*Effect of lamellarins on rhodamine 123 accumulation in multidrug resistant-cells*

Figure 3 shows the effects of increasing concentrations of verapamil or lamellarin I on the intracellular accumulation of rhodamine 123 in Lo Vo and P-gp-positive Lo Vo/Dx cells after 30 min incubation with 20 $\mu$M rhodamine 123. Compounds at toxic concentrations could be tested in accumulation studies because incubation time is not sufficient for cell death to occur. As shown in Figure 3, rhodamine 123 accumulates in parental Lo Vo cells, but not in multidrug-resistant Lo Vo/Dx cells. Verapamil and lamellarin I increased the intracellular concentration of rhodamine 123 in Lo Vo/Dx cells in a dose-dependent manner, and raised it to the level observed in the sensitive cells. At identical concentration, lamellarin I increased

**Figure 2** Dose-dependent effect on the *in vitro* growth of multidrug-resistant P388/Schabel cells by doxorubicin alone (-□-) or in the presence of 20 (-●-) or 2 $\mu$M (-○-) verapamil or 2 (-△-) or 0.2 (-▲-) $\mu$M lamellarin I. As a reference the growth of P388 parental cells is displayed (-■-). Cell proliferation is represented as percentage of control cell growth in cultures containing no drugs. Each point represents the mean of triplicates; s.d. values were always lower than 10% and are omitted for clarity.

**Figure 3** Effect of verapamil (○) and lamellarin I (●) on the accumulation of rhodamine 123 by multidrug-resistant Lo Vo/Dx cells in co-treatment conditions. Approximately $10^5$ cells per well were used in the assay. Rhodamine 123 accumulation in Lo Vo parental cells is shown by a dashed line. Each point represents the mean of four determinations ± s.d.

**Table II** Gains of sensitivity (GS) to doxorubicin, daunorubicin and vinblastine for multidrug-resistant P388/Schabel cells, obtained with different concentrations of verapamil and lamellarin I

| | | *Mean GS ± s.d.[a] at μM RMA* | | | |
|---|---|---|---|---|---|
| *Antitumoral drug* | *RMA* | *0.2* | *1* | *2* | *20* |
| Doxorubicin | Verapamil | 1.0 ± 0.4 | 4.1 ± 0.7 | 12.1 ± 4.6 | 101 ± 17 |
| | Lamellarin I | 2.7 ± 0.5 | 46.8 ± 8.1 | 105 ± 11 | * |
| Daunorubicin | Verapamil | ND | 5.6 ± 0.78 | 12.4 ± 1.7 | 94.5 ± 21 |
| | Lamellarin I | ND | 49 ± 5.3 | 198 ± 27 | * |
| Vinblastine | Verapamil | ND | 3.2 ± 0.5 | 16.5 ± 3.9 | 220 ± 31 |
| | Lamellarin I | ND | 63 ± 7.1 | 210 ± 25 | * |

[a] Mean of 3 – 8 determinations in triplicate ± s.d. ND, not determined; RMA, resistance modifier agent. * > 50% growth inhibition by RMA alone. For calculation of GS see Materials and methods.

steady-state rhodamine 123 accumulation to a higher level than verapamil. The maximal enhancement in accumulation in resistant cells was obtained using 40 and 10 μM of verapamil and lamellarin I respectively, and corresponds to the level of rhodamine 123 measured in Lo Vo cells. Neither of the agents modulated rhodamine 123 accumulation in the sensitive cells.

### Discussion

MDR remains a main obstacle to long-term successful cancer chemotherapy. The clinical need to overcome this resistance has fuelled the search for new cytotoxic drugs active on MDR cells, as well as of compounds capable of blocking *in vivo* the activity of P-gp. Thus far, reversal of MDR by a broad spectrum of compounds such as calcium channel blockers, calmodulin inhibitors, local anaesthetics and synthetic isoprenoids, has been described. However, *in vivo* studies have been disappointing because MDR modulators often reveal intolerably high toxic side-effects in humans and on the other hand clinically relevant concentrations of MDR modifiers can only rarely be achieved (Raderer and Sheithauer, 1993).

The results of this study suggest that lamellarins may be useful in the treatment of multidrug-resistant tumours by means of two independent mechanisms of action: cytotoxicity against cancer cells and enhancement of the cytotoxicity of doxorubicin against MDR cells, restoring in them the levels of sensitivity to those of the parental cells.

Five of the lamellarins tested: lamellarins D-triacetate, K, K-triacetate, M and N-triacetate display considerable cytotoxic activity against all the tumour cell lines tested. Two of them, lamellarins D-triacetate and K-triacetate, exhibit a higher activity on A549 human lung carcinoma cells. The anti-tumour activity of the five lamellarins mentioned on multidrug-resistant cell lines is similar to that obtained on the corresponding parental cell lines. The mode of action of the cytotoxicity of lamellarin alkaloids is still unknown, but it seems obvious that their level of activity is not affected by P-gp. This could be due to two different reasons: either lamellarins are not extruded by P-gp, or they inhibit P-gp pumping activity, therefore allowing an effective intracellular concentration of lamellarins in multidrug-resistant cells. Our findings that all the lamellarins tested are able to increase the intracellular accumulation of rhodamine 123 in multidrug-resistant cells (results not shown) support the last speculation.

Although a clear correlation between structure and cytotoxic activity of the lamellarins tested cannot be established, it seems that an increase in the number of methylations and/or methoxylations cause a decrease in the antitumoral activity of the compounds. This is in agreement with data from Toffoli *et al.* (1995), who have recently reported that the presence of methoxy groups in the verapamil molecule structure prevented cytotoxicity when the verapamil analogues were used alone on a human colon cell line.

After determination of cytotoxicity, the different lamellarins were tested for chemosensitisation at non-toxic concentrations. In the primary screening, lamellarin I was the most potent of all the lamellarins tested for both chemosensitisation to doxorubicin-mediated inhibition of P388/Schabel cell growth, and restoration of the retention of rhodamine 123 in Lo Vo/Dx cells.

Lamellarin I completely reverses doxorubicin, daunorubicin and vinblastine resistance in P388/Schabel cells at 2 μM. Verapamil can completely reverse doxorubicin and vinblastine resistance, but not daunorubicin resistance, at non-toxic concentrations. The different pattern of chemosensitisation by verapamil and lamellarin I for doxorubicin, daunorubicin and vinblastine could be due to the existence of different drug binding-transport sites on P-gp for different drugs or groups of drugs, as previously suggested by Jachez *et al.* (1993*b*), and it could suggest that verapamil and lamellarin I possess different efficiencies at inhibiting those sites. Such preferences have been described previously for a series of derivatives of the natural macrolide antibiotic FK-506 (Jachez *et al.*, 1993*a*).

Reduced intracellular drug accumulation, expression of P-gp and reversibility by several classes of membrane-active agents that increase the intracellular drug accumulation characterise the MDR phenotype. Rhodamine 123 has proved to be a helpful tool for the evaluation of the activities of various molecules known or supposed to be RMAs (Pourtier-Manzanedo *et al.*, 1993). Rhodamine 123, which selectively locates in mitochondria, is effluxed more efficiently by MDR cells, and this efflux can be inhibited by verapamil and other RMAs (Twentyman *et al.*, 1994). Lamellarin I is able to increase rhodamine 123 retention in the MDR Lo Vo/Dx cells to a level similar to that of the drug-sensitive Lo Vo cell line. This effect was obtained at a lower concentration than that needed when the reference substance, verapamil, is employed. Measurement of rhodamine 123 accumulation yields a direct measurement of the inhibition of P-gp function. The increase in accumulation of rhodamine 123 in multidrug-resistant cells after addition of lamellarin, supports the hypothesis that this compound causes a modulation of resistance by inhibiting the pump function of P-gp.

Although it is difficult to find structural features that are common to a large number of chemosensitisers, it has been suggested that RMAs are hydrophobic, contain two or more planar aromatic rings, and a tertiary nitrogen (Zamora *et al.*, 1988; Pearce *et al.*, 1989). The structure of lamellarins fits this profile.

In conclusion, the potential use of lamellarins for the treatment of multidrug-resistant tumours may follow two different approaches: at toxic concentrations they can be used as antitumoral drugs active on the resistant tumours, and at non-toxic concentrations they can be employed as reversing agents, that is, compounds able to potentiate the cytotoxic activity of other antitumoral drugs such as doxorubicin.

The testing of additional lamellarins may disclose the existence of other agents with either a higher cytotoxic activity on tumour cells, or a more potent modulating activity on multidrug-resistant cells and low, if any, cytotoxic activity, which could make their use preferable in future clinical trials. Further *in vitro* as well as *in vivo* experiments will indicate whether lamellarins can be of important clinical use as antitumoral drugs and/or in reversing multidrug resistance.

**Acknowledgements**
Thanks are due to P Rol for her excellent technical assistance and to I Núñez de Castro and MA Medina for their critical reading of the manuscript.

### References

ANDERSEN RJ, FAULKNER DJ, CUN-HENG H, VAN DUYNE GD AND CLARDY J. (1985). Metabolites of the marine prosobranch mollusc *Lamellaria* sp. *J. Am. Chem. Soc.*, **107**, 5492–5495.

ARCECI RJ. (1993). Clinical significance of P-glycoprotein in multidrug resistance malignancies. *Blood*, **81**, 2215–2222.

BECK WT AND QIAN XD. (1992). Photoaffinity substrates for P-glycoprotein. *Biochem. Pharmacol.*, **43**, 89–93.

BENCHIMOL S AND LING V. (1994). P-glycoprotein and tumor progression. *J. Natl Cancer Inst.*, **86**, 814–815.

BIEDLER JL. (1994). Drug resistance: genotype versus phenotype. Thirty-second G.H.A. Clowes Memorial Award Lecture. *Cancer Res.*, **54**, 666–678.

CARROLL AC, BOWDEN BF AND COLL JC. (1993). Studies of Australian ascidians, *Didemnum* sp. *Aust. J. Chem.*, **46**, 489–501.

CONFORTI G, CODEGONI AM, SCANZIANI E, DOLFINI E, DASDIA T, CALZA M, CANIATTI M AND BROGGINI M. (1995). Different vimentin expression in two clones derived from a human colocarcinoma cell line (Lo Vo) showing different sensitivity to doxorubicin. *Br. J. Cancer*, **71**, 505–511.

GANAPATHI R AND GRABOWSKI D. (1983). Enhancement of sensitivity to doxorubicin in resistant P388 leukemia cells by the calmodulin inhibitor trifluoperazine. *Cancer Res.*, **43**, 3696–3699.

GOTTESMAN MM AND PASTAN I. (1993). Biochemistry of multidrug resistance mediated by the multidrug transporter. *Annu. Rev. Biochem.*, **62**, 385–427.

GRANDI M, GERONI C AND GIULIANI FC. (1986). Isolation and characterization of a human colon adenocarcinoma cell line resistant to doxorubicin. *Br. J. Cancer*, **54**, 515–518.

GRANDI M, YOUNG C, BELLINI O, GERI O, MUINDI J AND GIULIANI F. (1987). Pleiotropic multidrug resistant Lo Vo, P388 and I-407 cell lines have an increased tubulovesicular compartment. *Proc. Am. Assoc. Cancer Res.*, **28**, 279.

JACHEZ B, BOESCH D, GRASSBERGER MA AND LOOR F. (1993*a*). Reversion of the P-glycoprotein-mediated multidrug resistance of cancer cells by FK-506 derivatives. *Anti-Cancer Drugs*, **4**, 223–229.

JACHEZ B, NORDMANN R AND LOOR F. (1993*b*). Restoration of taxol sensitivity of multidrug-resistant cells by the cyclosporine SDZ PSC 833 and the cyclopeptolide SDZ 280–446. *J. Natl Cancer Inst.*, **85**, 478–483.

KARTNER N, EVERNDEN-PORELLE D, BRADLEY G AND LING V. (1985). Detection of P-glycoprotein in multidrug-resistant cell lines by monoclonal antibodies. *Nature*, **316**, 820–823.

KELLER RP, ALTERMATT HJ, NOOTER K, POSCHMANN G, LAISSUE JA, BOLLINGER P AND HIESTAND PC. (1992). SDZ PSC 833, A non-immunosuppressive cyclosporine: its potency in overcoming P-glycoprotein-mediated multidrug resistance of murine leukemia. *Int. J. Cancer*, **50**, 593–597.

LINDQUIST N, FENICAL W, VAN DUYNE G AND CLARDY J. (1988). New alkaloids of the lamellarin class from the marine ascidians *Didemnum chartaceum* (Sluiter, 1909). *J. Org. Chem.*, **53**, 4570–4574.

LING V. (1982). Genetic basis of drug resistance in mammalian cells. In *Drug and Hormone Resistance in Neoplasia*, Bruchovski N and Goldie JM (eds), Vol. 1 pp. 1–19, CRC Press: Miami, FL.

LOOR F, BOESCH D, GAVERIAUX C, JACHEZ B, POURTIER-MANZANEDO A AND EMMER G. (1992). SDZ 280–446, a novel semi-synthetic cyclopeptolide: in vitro and in vivo circumvention of the P-glycoprotein mediated tumour cell multidrug resistance. *Br. J. Cancer*, **65**, 11–18.

McBURNEY MW AND WHITMORE GF. (1974). Isolation and characterization of folate deficient mutants of Chinese hamster cells. *Cell*, **2**, 173–182.

MILLER RL, BUKOWSKI RM, BUDD GT, PURVIS J, WEICK JK, SHEPARD K, MIDHA KK AND GANAPATHI R. (1988). Clinical modulation of doxorubicin resistance by the calmodulin-inhibitor, trifluoperazine: A phase I/II trial. *J. Clin. Oncol.*, **6**, 880–888.

MIYAMOTO K, INOKO K, WAKUSAWA S, KAJITA S, HASEGAWA T, TAKAGI K AND KOYAMA M. (1993). Inhibition of multidrug resistance by a new staurosporine derivative, NA-382, *in vitro* and *in vivo*. *Cancer Res.*, **53**, 1555–1559.

MOSMANN T. (1983). Rapid colorimetric assay for cellular growth and survival: application to proliferation and cytotoxicity assays. *J. Immunol. Methods*, **65**, 55–63.

NOOTER K AND HERWEIJER H. (1991). Multidrug resistance (*mdr*) genes in human cancer. *Br. J. Cancer*, **63**, 663–669.

OZOLS RF, CUNNION RE, KLEKER RW, HAMILTON TC, OSTCHEGA Y, PARILLO JE AND YOUNG RC. (1987). Verapamil and doxorubicin in the treatment of drug-resistant ovarian cancer patients. *J. Clin Oncol.*, **5**, 641–647.

PATEL NH AND ROTHENBERG ML. (1994). Multidrug resistance in cancer chemotherapy. *Invest. New Drug*, **12**, 1–13.

PEARCE HL, SAFA AR, BACH NJ, WINTER MA, CIRTAIN MC AND BECK WT. (1989). Essential features of the P-glycoprotein pharmacophore as defined by a series of reserpine analogs that modulate multidrug resistance. *Proc. Natl Acad. Sci. USA*, **86**, 5128–5132.

PENNOCK GD, DALTON WS, ROESKE WR, APPLETON CP, MOSLEY K, PLEZIA P, MILLER TP AND SALMON SE. (1991). Systemic toxic effects associated with high-dose verapamil infusion and chemotherapy administration. *J. Natl Cancer Inst.*, **83**, 105–110.

POURTIER-MANZANEDO A, DIDLER AD, MULLER CD AND LOOR F. (1993). SDZ PSC 833 and SDZ 280–446 are the most active of various resistance-modifying agents in restoring rhodamine-123 retention within multidrug resistant P388 cells. *Anti-Cancer Drugs*, **3**, 419–421.

QUESADA AR, BARBACID MM, MIRA E, ARACIL M AND MARQUEZ G. (1996). Chemosensitization and drug accumulation assays as complementary methods for the screening of multidrug resistance reversal agents. *Cancer Lett.*, **99**, 109–114.

RADERER M AND SHEITHAUER W. (1993). Clinical trials of agents that reverse multidrug resistance. *Cancer*, **72**, 3553–3563.

RAMU A, SPANIER R, RAHAMIMOFF H AND FUKS Z. (1984). Restoration of doxorubicin responsiveness in P388 murine leukaemia cells. *Br. J. Cancer*, **50**, 501–507.

THOMSON LA AND BARKER RM. (1973). Isolation of mutants of cultured mammalian cells. *Methods Cell Biol.*, **6**, 209–281.

TOFFOLI G, SIMONE F, CORONA G, RASCHACK M, CAPPELLETTO B, GIGANTE M AND BOIOCCHI M. (1995). Structure–activity relationship of verapamil analogs and reversal of multidrug-resistance. *Biochem. Pharmacol.*, **50**, 1245–1255.

TSURUO T, IIDA H, TSUKAGOSHI S AND SAKURAI Y. (1981). Overcoming of vincristine resistance in P388 leukemia *in vivo* and *in vitro* through enhanced cytotoxicity of vincristine and vinblastine by verapamil. *Cancer Res.*, **41**, 1967–1972.

TSURUO T, IIDA H, NOJIRI M, TSUKAGOSHI S AND SAKURAI Y. (1983). Circumvention of vincristine and doxorubicin resistance *in vitro* and *in vivo* by calcium influx blockers. *Cancer Res.*, **43**, 2905–2910.

TWENTYMAN PR. (1988). Modification of cytotoxic drug resistance by non-immunosuppressive cyclosporins. *Br. J. Cancer*, **57**, 254–258.

TWENTYMAN PR AND BLEEHEN NM. (1991). Resistance modification by PSC-833 a novel non-immunosuppressive cyclosporin A. *Eur. J. Cancer*, **27**, 1639–1642.

TWENTYMAN PR, RHODES T AND RAYNER S. (1994). A comparison of Rhodamine 123 accumulation and efflux in cells with P-glycoprotein-mediated and MRP-associated multidrug resistance phenotype. *Eur. J. Cancer*, **30A**, 1360–1369.

ZAMORA JM, PEARCE HL AND BECK WT. (1988). Physical–chemical properties shared by compounds that modulate multidrug resistance in human leukemic cells. *Mol. Pharmacol.*, **33**, 454–462.